# Neutrophil to lymphocyte ratio as a predictor for diagnosis of early Periprosthetic joint infection

**DOI:** 10.1186/s12891-020-03704-5

**Published:** 2020-10-27

**Authors:** Bao-Zhan Yu, Jun Fu, Wei Chai, Li-Bo Hao, Ji-Ying Chen

**Affiliations:** 1 Department of Orthopaedics, Chinese People’s Liberation Army General Hospital, 28 Fuxing Road, Beijing, 100853 Haidian District China; 2 Department of Orthopaedics, Bao Ding GemFlower Eastern Hospital, Bao Ding, Hebei, P. R. China

**Keywords:** Neutrophil to lymphocyte ratio, Early periprosthetic joint infection, C-reactive protein, Interleukin-6

## Abstract

**Background:**

Periprosthetic joint infection (PJI) is a catastrophic complication after total knee or hip arthroplasty. The diagnosis of PJI is very difficult, especially in the early postoperative period. The value of the neutrophil to lymphocyte ratio (NLR) is useful for diagnosing infectious diseases. The objective of this study was to investigate the accuracy of the NLR for the diagnosis of early PJI after total knee or hip arthroplasty.

**Methods:**

We retrospectively evaluated consecutive primary total knee or hip arthroplasty and identified the patients who readmitted within the first 90 days postoperatively between January 2011 and October 2018.There were 20 cases diagnosed early PJI and 101 uninfected cases on the basis of the modified Musculoskeletal Infection Society (MSIS) criteria. The serum parameters including C-reactive protein (CRP), erythrocyte sedimentation rate (ESR), white blood-cell (WBC) count, NLR and interleukin-6 (IL-6) were compared between the two groups. Receiver operating characteristic curves were generated to estimate the optimal cutoff values for each parameter. The sensitivity, specificity, positive predictive value and negative predictive value for each parameter were calculated.

**Results:**

The CRP, ESR, WBC, NLR and IL-6 values were all significally higher in the infected group than the uninfected group. The median of CRP was 66.6 mg/l in the infected group and 8.6 mg/l in the uninfected group (*p* < 0.001). The median of ESR was 34.8 mm/hr. in the infected group and 17.4 mm/hr. in the uninfected group (p < 0.001). In the infected group and uninfected group, the median of WBC was 8.2X10^9^ /L and 6.1 X10^9^ /L (*p* = 0.002), respectively; while the median of NLR was 5.2 and 2.1 (*p* < 0.001). The median of IL-6 was 46 pg/ml and 6.4 pg/ml (p < 0.001),respectively. The best parameter for the diagnosis of early PJI was IL-6 (AUC = 0.814) followed by the NLR (AUC =0.802), CRP (AUC =0.793), ESR (AUC =0.744) and WBC (AUC = 0.632).

**Conclusions:**

This study is the first to show that NLR values are more accurate than CRP and may be considered as useful parameters for the diagnosis of early PJI because it is a cheap and convenient parameter to be calculated in daily practice without extra costs.

## Background

Periprosthetic joint infection (PJI) is a disastrous complication after total knee or hip arthroplasty with high morbidity and mortality. The early and accurate diagnosis of PJI is of great clinical significance for the treatment of PJI [1, 2]. A less invasive procedure to retain the prosthetic components may be adopted in the early postoperative stage. However, the diagnosis of PJI is very difficult due to the lack of an absolute diagnostic test. It remains even more challenging for the diagnosis of early PJI because the normal periincisional swelling and erythema make it difficult to distinguish an early postoperative infection from the normal postoperative course.

It is essential to diagnose early PJI on the basis of a combination of clinical judgment, blood testing, synovial fluid aspiration, microbiologic and histopathologic inspections as well as imaging. There are various imaging techniques used in the assessment of PJI including radiographs, CT, MRI, ultrasound and nuclear medicine examinations. The ultrasonography (US) and MRI may be valuable tool for diagnosis of early PJI, which are characterized by soft tissue swelling, fluid collections and presence of sinus around the joint. However, there are very few literatures regarding for the diagnosis of early PJI because of lack of a suitable control group as the truly aseptic revisions are rare in the early postoperative period. Kim et al. [3] found that the serum CRP may be a wonderful screening test in the workup of early PJI after TKA. However, Bedair et al. [4] reported that the sensitivity of CRP in serum for diagnosing early PJI was only 53%. The synovial fluid aspiration is also an excellent test in the workup of early PJI. The sensitivity and specificity of synovial WBC counts are approximately 95–98.9% and 91–100% respectively [3–5]. But the synovial fluid aspiration is an invasive operation and sometimes it is very hard to obtain synovial fluid even though repeat joint aspiration, especially for the hip joint.

Among the various kinds of tests, it is very critical to find a simple and practical marker for diagnosing early PJI. The serum WBC count has very little utility due to the very low sensitivity as markers of PJI [6]. In recently, the value of the neutrophil to lymphocyte rate (NLR) that is obtained from the absolute neutrophil and absolute lymphocyte counts of a complete blood count is routinely used to predict outcomes as an available marker in oncology, cardiovascular diseases and infections [7–9]. Yombi et al. [10] found that the NLR may be potentially a better biomarker for the detection of early PJI after TKA because it had a faster normalization than CRP. However, further studies are still required to determine the conclusions through a comparative study. To our knowledge, there were no related studies. Therefore, the purpose of this study was to determine the accuracy of the NLR in the diagnosis of early PJI.

## Methods

After Institutional Review Board approval for this study was obtained, we retrospectively reviewed the cases of consecutive primary total knee or hip arthroplasty in our institutions between January 2011 and October 2018. A total of 245 cases readmitted within the first 90 days postoperatively were identified. These patients were admitted to hospital again in 90 days for the following reasons: (1) staged bilateral total knee or hip arthroplasty, (2) dislocation, (3) patellar ligament rupture, (4) persistent wound drainage, (5) fever, (6) fracture, (7) erythema,(8) superficial infection, (9) postoperative infection was suspected.

Blood samples for CRP, ESR, IL-6 and other markers were taken on the morning after readmission for all patients and were then sent to the medical laboratory center for testing as soon as possible. All data including CRP, ESR, white blood cell (WBC) count, IL-6, neutrophil count and lymphocyte count were obtained from electronic medical records by manual chart review. The NLR was obtained as the absolute neutrophil count divided by the absolute lymphocyte count.

The aspirations were performed for these patients which resulted from symptoms such as persistent wound drainage,fever, erythema, superficial infection and postoperative infection being suspected. The synovial fluids were used for bacterial culture, a leukocyte esterase (LE) strip test and synovial fluid analysis (white blood-cell [WBC] count and polymorphonu-clear leukocyte [PMN] percentage).

These inflammatory arthritis, such as rheumatoid arthritis, ankylosing spondylitisetc were excluded in order to ruling out interference with other possible preconditions associated with elevated inflammatory markers. Also excluded were 1) Patients with superficial infection (24cases); 2) postoperative fever (12cases, because of upper-respiratory infection, urinary tract infection); 3) a history of malignancy (4cases); 4) missing critical data (including CRP,IL-6 or ESR).

**Fig. 1:**
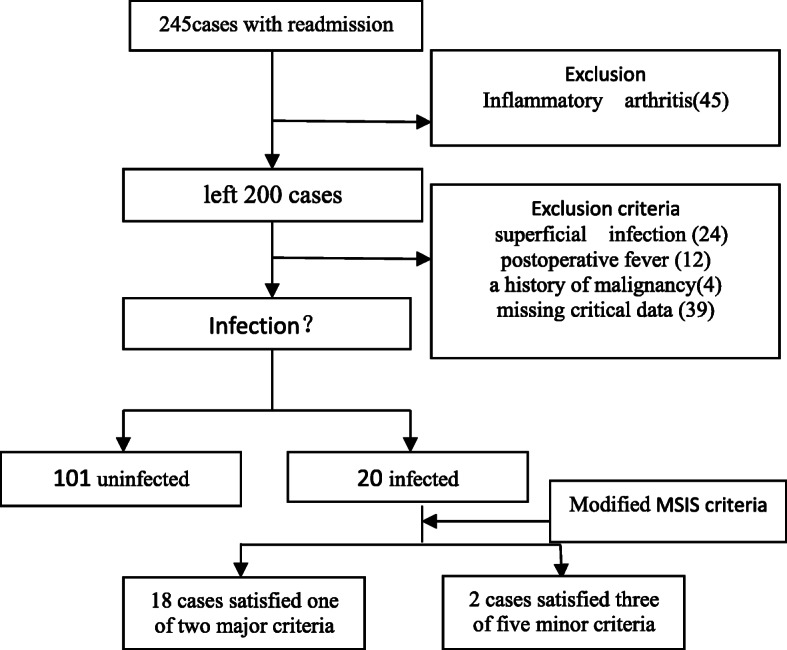
Flowchart

These exclusions left 121 cases were analyzed. There were 20 infected cases and 101 uninfected according to the Musculoskeletal Infection Society (MSIS) criteria [11] (Table 1). Eighteen cases that satisfied one of two major criteria: a sinus tract communicating with the prosthesis (16), two positive periprosthetic cultures with phenotypically identical organisms (2); 2 cases that satisfied three of five minor criteria: a single positive intraoperative periprosthetic tissue culture, elevated synovial fluid white blood cell (WBC) count and a positive histological analysis of periprosthetic tissue (> 5 neutrophils per high power field) (Fig. 1).

**Table 1:** The Modified Musculoskeletal Infection Society (MSIS) criteria

PJI is present if one of two major criteria or three of five minor criteria exists:			
Major criteria	1. A communicating sinus tract with the prosthesis; or		
	2. Two positive periprosthetic cultures with phenotypically identical organisms		
Minor criteria	Having three of the following minor criteria:	Acute PJI (< 90 days)	Chronic PJI (> 90 days)
	1. Serum CRP or ESR	ESR: no threshold	ESR: >30mm/h
	2. Synovial fluid WBC count or	10,000 cells/μL	3000 cells/μL
	Changes in leukocyte esterase test strip	+ or ++	+ or ++
	3. Synovial fluid PMN %	90%	80%
	4. Positive histologic analysis of the periprosthetic tissue	> 5 neutrophils per high-power field (× 400)	> 5 neutrophils per high-power field (× 400)

*ESR* Erythrocyte sedimentation rate, *CRP* C-reactive protein, *WBC* White blood cell count, *PMN* Neutrophils differential

There were 19 cases that underwent debridement, antibiotics and implant retention (DAIR) and one case was managed with one-stage revision in the infected group. Three cases in the infected group failed and no cases were diagnosed and reoperated for PJI in the uninfected group with at least 1-year follow-up. Twenty four cases of superficial infection were also analyzed. There was no case that were diagnosed and reoperated for PJI with at least 1-year follow-up.(Table 2).

**Table 2:** Detailed characteristics of patients with superficial infection

Patient	Sex(male/M female/F)	Joint (knee/hip)	Primary disease	Interval time readmission(day)	Follow-up time(months)
1	F	*Knee*	OA	15	76
2	F	*Knee*	OA	30	71
3	F	*Knee*	OA	21	56
4	M	*Knee*	OA	23	53
5	M	*Knee*	OA	11	48
6	F	*Knee*	OA	7	35
7	F	knee	OA	45	32
8	F	Knee	OA	25	28
9	M	Hip	ONFH	14	36
10	F	Hip	ONFH	8	27
11	M	Hip	ONFH	5	24
12	M	Hip	ONFH	14	24
13	F	*Knee*	OA	10	18
14	F	*Hip*	OA	11	29
15	F	Hip	DDH	9	31
16	M	*Knee*	OA	7	24
17	M	*Knee*	OA	15	25
18	F	*Hip*	OA	8	22
19	M	Hip	ONFH	6	24
20	F	Hip	DDH	23	18
21	F	Hip	DDH	13	17
22	F	Knee	OA	17	15
23	F	Knee	OA	8	15
24	M	Knee	OA	12	12

*OA* Osteoarthritis, *ONFH* Osteonecrosis of the Femoral Head, *DDH* Developmental dysplasia hip

### Statistical analysis

The categorical variables were assessed using chi-square tests and parametric data were analyzed using t tests. The results of the diagnostic tests in the two groups were compared using the Mann-Whitney test. Statistical significance was considered as a *p* < 0.05. The whole statistical analysis were performed with use of the statistical software packages R (The R Foundation) and Empower (X&Y Solution). The Receiver operating characteristic (ROC) curves and area under the curve (AUC) were calculated to examine each parameter for the evaluation of early PJI. The Youden index was used to determine the optimal threshold for each parameter as a diagnostic tool for early PJI. The sensitivity and specificity of each parameter were calculated.

## Results

There were 20 cases in infected group and 101 cases in uninfected group based on the MSIS criteria. There was no significantly different in age, time interval, BMI, joint (hip or knee) between two groups. The characteristics of each cohort are shown in (Table 3).

**Table 3:** Patient characteristics

Variable	Infected (n=20)	Uninfected (n=101)	*P-*value
Sex			0.03
Male	13 (65%)	22(21.78%)	
Female	7 (35%)	79 (78.22%)	
Age	68.2 (44-82)	64.9 (20-84)	0.968
BMI (kg/m^2^)	28.43 (18.37-48.68)	27.74(18.59-38.35)	0.734
Joint			0.562
Hip	3 (15%)	25 (24.75%)	
Knee	17 (85%)	76 (75.25%)	
Time	45(7-87)	40(5-90)	0.132

*BMI* Body mass index

The median of CRP was 66.6 mg/l ((interquartile range [IQR], 0.5 to 208.9) in the infected group and 8.6 mg/l ([IQR], 0.5 to 79.4) in the uninfected group (*p* < 0.001). The median of ESR was 34.8 mm/hr. ([IQR], 7 to 78) in the infected group and 17.4 mm/hr. ([IQR], 3 to 79) in the uninfected group (p < 0.001). In the infected group and uninfected group, the median of WBC was 8.2X10^9^ /L (IQR, 4.2 to 28.1 X10^9^ /L) and 6.1 X10^9^ /L (IQR, 3.0 to 14.8 X10^9^ /L) (*p* = 0.002), respectively; while the median of NLR was 5.2 ([IQR], 0.9 to 18.8) and 2.1([IQR], 0.8 to 6.4) (*p* < 0.001). The median of IL-6 was 46 pg/ml ([IQR], 1.5 to 347) and 6.4 pg/ml ([IQR], 1.5 to 40.9) (*p* < 0.001), respectively. (Table 4).

**Table 4:** Median values of diagnostic measures between infected and uninfected group

Variable	Infected (n=20)	Uninfected (n=101)	*P-*value
CRP	66.6 (0.5-208.9)	8.6 (0.5-79.4)	<0.001
ESR	34.8(7.0-78.0)	17.4(3.0-79.0)	<0.001
WBC	8.2 (4.2-28.1)	6.1 (3.0-14.8)	0.0024
NLR	5.2 (0.9-18.8)	2.1 (0.8-6.4)	<0.001
IL-6	46.0 (1.5-347.0)	6.4 (1.5-40.9)	<0.001

*CRP* C-reactive protein, *ESR* Erythrocyte sedimentation rate, *WBC* White blood cell count, *IL-6* Interleukin-6, *NLR* Neutrophil-to-lymphocyte ratio

**Fig. 2:**
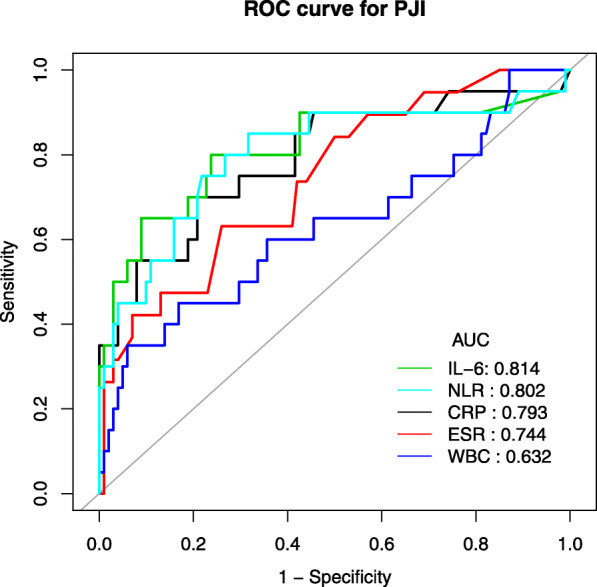
The ROC curves for C-reactive protein (CRP), erythrocyte sedimentation rate (ESR), white blood-cell count (WBC), neutrophil to lymphocyte ratio (NLR) and interleukin-6(IL-6) in predicting early PJI

The ROC curve analysis showed the IL-6 was the best test for diagnosing early PJI (AUC =0.814) followed by the NLR (AUC =0.802), CRP (AUC =0.793), ESR (AUC =0.744) and WBC (AUC = 0.632) (Fig. 2). We identified the cutoff values for the IL-6 at 8.07 pg/ml. The sensitivity, specificity, positive predictive value (PPV), and negative predictive value (NPV) was 80, 76.2, 40, and 95.1%, respectively. With the calculated cutoff value of NLR set at 2.13, the sensitivity, specificity, positive predictive value (PPV), and negative predictive value (NPV) was 85, 68.3, 34.7, and 95.8%, respectively (Table 5).

**Table 5:** Values of serum parameters in predicting PJI

	AUC	(95%CL)	Threshold	Sensitivity	Specificity	PPV	NPV
CRP	0.793	0.668-0.918	9.27mg/l	0.700	0.792	0.400	0.930
ESR	0.744	0.621-0.867	22mm/hr	0.632	0.740	0.316	0.914
WBC	0.632	0.479-0.784	8.91x10^9^/L	0.350	0.941	0.538	0.880
NLR	0.802	0.674-0.930	2.13	0.850	0.683	0.347	0.958
IL-6	0.814	0.683-0.945	8.07pg/ml	0.800	0.762	0.400	0.951

*AUC* Area of under the receiver operating characteristic curve, *PPV* Positive predictive value, *NPV* Negative predictive value

## Discussion

It is very difficult to identify an early postoperative infection after total knee or hip arthroplasty because there is no an absolute diagnostic criteria. In this study, we determined interleukin-6(IL-6) in serum had the highest accuracy as a marker for diagnosing early PJI, followed by the NRL, the C-reactive protein, the erythrocyte sedimentation rate, and the white blood cell count.

Interleukin-6 is an inflammatory cytokine produced by activated monocytes and macrophages and induces to product some major acute-phase proteins, including CRP [12]. Some studies have showed that the IL-6 in serum was a valuable marker for diagnosis of PJI. Di Cesare et al. [13] showed serum IL-6 was more accurate marker than either ESR or the CRP level for diagnosing chronic PJI. A meta-analysis [14] published that IL-6 in serum was a potential superior diagnostic test compared to the conventional CRP and ESR, with the pooled sensitivity and specificity of 97 and 91%, respectively. At present there were no reports about IL-6 in serum as a useful marker for diagnosis of early PJI. Maniar et al. [15] found the normal trajectory of IL-6 had a more rapid increase and quicker return to normal than CRP after uncomplicated TKA. Although the author could not comment specifically on the value in diagnosing PJI, any deviation of IL-6 from a known normal trajectory could promptly make a decision to perform knee aspiration to diagnose early PJI. However, in our study, when we identified the cutoff values for the IL-6 at 8.07 pg/ml, the sensitivity, specificity was 80, 76.2%, respectively. IL-6 in serum may be a useful marker for diagnosing early PJI. This is the first to assess the use of serum IL-6 to diagnose early PJI.

The serum IL-6 was not often detected because of expensive costs. Interestingly, another indicator, NLR performed very well and had the high AUC (0.802). The accuracy of the value of NLR was less than IL-6 but greater than CRP. It may be more suitable for screening to diagnose early PJI because it is convenient to be obtained in daily practice without extra costs.

The value of the NRL had been shown a significant association with infection. Dogruel et al. [16] demonstrated there were statistically significant correlations between NLR and prolonged hospital stay and postoperative antibiotic doses for the treatment of odontogenic infection. Josse et al. [17] reported that the values of NLR ≥2.3 in the pre-operation may be an independent predictor for major operative complications such as wound infection in patients undergoing colorectal surgery. Bolat et al. [18] investigated the relationship between NLR and early postoperative infection as a complication of penile prosthesis implantation (PPI). And the values of NLR may be a valid laboratory predictor for early postoperative infectious complications for patients who went through PPI. De Jager et al. [19] described the NLR had the highest AUC of 0.73 and differed significantly from the CRP level of 0.62. The NLR was better value in predicting bacteremia than routine parameters like CRP. Yombi et al. [10] reported that the NLR had a distribution trajectory in a standard post-operative period after TKA, and returned to normal values faster than CRP. It was potentially a better biomarker to follow post-operative inflammation or early infection after TKA. On the basis of our data, with the calculated threshold of NLR set at 2.13, the sensitivity, specificity, positive predictive value (PPV), and negative predictive value (NPV) was 85, 68.3, 34.7, and 95.8%, respectively. To our knowledge, this is the first study to assess the NLR in diagnosing early PJI.

Although there were some literatures that elaborate possible explanations about the relation between elevated NLR and the development of [20–22], it remained unclear about the exact mechanism. The neutrophils play pivotal roles in the progression of the bacterial infection. The key cell types of the innate immune system as well as the first cellular line of defense against infection are neutrophils. Lymphocytes are involved in adaptive immune response. The physiological immune responses of circulating leukocytes to various stressful events has distinctive feature: increase in neutrophil count and decrease in lymphocyte count. An increase in neutrophils is an inflammatory reaction, particularly when caused by a bacterial infection [23]. Lymphocytopenia has also been described as a diagnostic marker of bacterial infection [24]. Therefore, the NLR is thought to have stronger discriminative power for predicting bacteremia compared to discrimination based on neutrophilia or lymphocytopenia alone. The most crucial finding of this study was that NLR may be a useful parameter for diagnosing early PJI.

There are some limitations of this study that should be considered. First, this is retrospective study and the inherent limitations exist. There may be some selection bias because several potential cases were excluded due to no record of the serum ESR, CRP, or IL-6. Although this study is a retrospective design, it may provide some useful information for diagnosing early PJI. Second, there is no consensus on definition of the early PJI. We defined 90 days as early PJI and shorter time needed to be detected. Finally, we recruited 121 cases to the study. Although the total sample size was relatively sufficient, there was a small sample size in infected cases that limited the statistical power of our conclusions. Therefore, our findings should be confirmed in a larger study and at multiple institutions.

## Conclusions

The findings of this study have shown that the IL-6 was the best test for diagnosis of early PJI. But it was not often detected because of expensive costs. However, the NLR that has the higher accuracy for the diagnosis of early PJI than CRP may be considered as a useful parameter for the diagnosis of early PJI because it is easy, cheap and convenient to be calculated in daily practice without extra costs. However, further studies evaluating the accuracy of NLR in more patients are needed to verify the findings of the present study.

## Data Availability

We do not wish to share our data, because some of the patient’s data regarding individual privacy, and according to the policy of our hospital, the data could not be shared with others without permission. An anonymised form of the data could be made available from the corresponding author upon reasonable request.

## Abbreviations

PJI: Periprosthetic joint infection
NLR: Neutrophil to lymphocyte ratio
TKA: Total knee arthroplasty
AUC: The area under the ROC curve
BMI: Body mass index
CRP: C-reactive protein
ESR: Erythrocyte sedimentation rate
IL-6: Interleukin-6
LE: Leukocyte esterase
MSIS: Musculoskeletal Infection Society
NPV: Negative predictive value
PMN%: Percentage of polymorphonuclear cells
PPV: Positive predictive value
ROC: Receiver operating characteristic
WBC: White blood cell
PPI: Penile prosthesis implantation
